# Progress in Gynæcology

**Published:** 1899-08-12

**Authors:** 


### PROGRESS IN GYNÆCOLOGY.

**The Use and Abuse of Pessaries.**—Montgomery,[1] in a paper on the use and abuse of pessaries, after referring to the increased popularity of surgical measures for retaining the uterus in its normal position, points out that many patients are unwilling to submit to operative procedures, even if a complete cure can be promised; and he says that the majority of these are enabled through the use of the pessary to secure complete relief while wearing it. After discussing the various displacements in which the pessary is most useful, he maintains it must be considered an abuse of that instrument if it is used under the following conditions: (1) When the patient suffers no inconvenience from the displacement; (2) when inflammatory or perimetric adhesions exist which preclude the possibility of maintaining the uterus in its normal situation; (3) in cases of large uteri with acute retroflexion, as in such cases the uterus is liable to slip behind the posterior bar and be crowded by it against the sacrum. Dr. J. W. Ballantyne[2] says that, notwithstanding the advance in the surgical treatment of displacements, inquiry at the various instrument makers showed: (1) A steady increase in the number of pessaries sold during the last 20 years; (2) a decrease in the varieties asked for, the most frequent being the ring and Hodge or their modifications, and occasionally a vaginal stem; (3) an almost complete absence of demand for intra-uterine stems. Medical men showed a marked tendency, after trying various forms of the Hodge pessary, to return to the Albert Smith modification in vulcanite; and after testing different kinds of rings to revert to the use of the simple rubber instrument containing a watch-spring. Dr. Ballantyne analysed the opinions expressed in 20 text-books on gynæcology published in the last 10 years. Of these eight authors were strongly in favour of this method of treating uterine displacements, five were strongly against it, and seven were critical, and even sceptical, without actually going so far as to condemn the method and banish the instruments. Different objections have been raised to the use of the pessary; there was the inconvenience, as they required attention by frequent douchings and occasional visits to the physician; they were also credited with being the cause of pruritus, vaginitis, ulceration, fistula, subinvolution, cancer, and septic inflammation of the uterus and tubes. Blondel[3] records an interesting case in which he removed with very great difficulty an old-fashioned cup pessary which had been in the vagina for 32 years; it had made a deep groove in the vaginal mucous membrane, almost perforating into the rectum; the woman had suffered for some time from an abundant fœtid sanious vaginal discharge. The supporters of the pessary claimed that any inconvenience or attention necessitated by the pessary was not to be compared with the trouble and expense attending an operation. Opinions varied with regard to the most suitable pessaries for the different conditions. In incomplete prolapse, when the perineum could be depended on, many recommended the india-rubber ring, or Hodge-Smith pessary with or without transverse bars, according as there was or was not some degree of cystocele. In complete prolapse, when the support of the pelvic floor was lost, the only possible pessary was the vaginal stem, with an abdominal belt, outside straps, and a perineal pad. The Zwanck and all instruments with hinges and screws were generally regarded as both unsatisfactory and dangerous. For anteflexion with well marked symptoms the use of the intra-uterine stem was considered justifiable; all recognised, however, the risks of sepsis, inflammation, and perforation of the uterus. In retroversions of the uterus most writers agreed that their correction by a pessary was followed by an amelioration or a total disappearance of the symptoms. The ring and Albert Smith pessaries were generally used for these conditions, the latter being carefully fitted to the size, shape, and curvature of the vagina.

**Chronic Pelvic Inflammation.**—Dr. Eden,[4] writing on this subject, says the treatment is either palliative or radical. The first degree of palliative treatment consists of prolonged rest in the recumbent position either in bed or on a couch, combined with hot douches, glycerine or ichthyol plugs, and blistering or counter irritation by the application of iodine to the anterior abdominal wall. If this plan has been tried without much success the next thing to do is to curette the uterus. This appears to act as follows: Foci of inflammation remain in the endometrium; and a certain amount of absorption occurring through lymphatic channels from these foci, the inflammatory process is thus kept alive. The uterus thus forms a source of continual reinfection of the deep parts. If you curette the uterus and cauterise the raw surface afterwards you will probably succeed in destroying the source of infection which has kept up the trouble, and you give the lesions in the deeper parts a chance of resolving. It is often necessary to curette a second time. If very little benefit arises from this the only thing left to do is to perform a radical operation. This consists either in removing the tubes and ovaries, or in removing the uterus and leaving the tubes and ovaries, or it may consist in removing the uterus, the tubes, and the ovaries. Dr. Eden thinks it is advisable in many cases to take away the uterus, and, if possible, leave one in both ovaries.

[1] International Med. Mag., Mar., 1899. [2] Scottish Med. and Surg. Jour., Mar., 1899. [3] Brit. Med. Jour., April 1, 1899. [4] Clin. Jour., Mar. 29, 1899.

